# An update of malaria infection and anaemia in adults in Buea, Cameroon

**DOI:** 10.1186/1756-0500-3-121

**Published:** 2010-04-30

**Authors:** Ebako N Takem, Eric A Achidi, Peter M Ndumbe

**Affiliations:** 1 Ministry of Health, BP 281, Buea, Cameroon; 2 Faculty of Health Sciences, University of Buea, Cameroon

## Abstract

**Background:**

Anaemia is caused by many factors in developing countries including malaria. We compared anaemia rates in patients with malaria parasitaemia to that of patients without malaria parasitaemia.

**Findings:**

A cross-sectional study was carried out from November 2007 to July 2008 in health units in Buea, Cameroon. Adult patients with fever or history of fever were included in the study. Information on socio-demographic variables and other variables was collected using a questionnaire. Malaria parasitaemia status was determined by microscopy using Giemsa stained thick blood smears. Haemoglobin levels were determined by the microhaematocrit technique.

The study population consisted of 250 adult patients with a mean age of 29.31 years (SD = 10.63) and 59.44% were females. 25.60% of the patients had malaria parasitaemia while 14.80% had anaemia (haemoglobin < 11 g/dl). Logistic regression revealed that those with malaria parasitaemia had more anaemia compared to those without malaria parasitaemia(OR = 4.33, 95%CI = 1.21-15.43, p = 0.02) after adjusting for age, sex, rural residence, socioeconomic status, use of antimalarials, use of insecticide treated nets(ITN) and white blood cell count.

**Conclusions:**

In adult patients with fever in this setting, malaria parasitaemia contributes to anaemia and is of public health impact. Our results also provide a baseline prevalence for malaria parasitaemia in febrile adults in health units in this setting.

## Background

Anaemia is frequent in developing countries and its aetiology is usually multifactorial. The most important factors that contribute to anaemia include parasitic infections, HIV infection, chronic inflammatory disorders, micronutrient deficiencies and genetic disorders [1-6]. The main parasitic infections include malaria and helminth infections [7-11].

Malaria related anaemia is associated with many factors which involve increased destruction and reduced production of red blood cells(RBCs) [12]. The relationship between malaria parasitaemia and haemoglobin has been well documented in previous studies in pregnant women [11,13-20]. Fewer previous studies in developing countries have involved non-pregnant adults [21-25]. Updating the role of malaria parasitaemia on anaemia in an era when the use of impregnated mosquito nets is being implemented will be of help to clinicians and public health specialists.

Our objective in this study was to compare anaemia rates in patients with malaria parasitaemia to that of patients without malaria parasitaemia in a population of adult patients with fever or history of fever in Buea, Cameroon.

## Methods

### Study population and design

The study was carried out in Buea in the South West of Cameroon. Buea is situated at the foot of Mount Cameroon and has an estimated population of 81 478 inhabitants (2008). The climate of Buea is of equatorial type with temperatures that range from 25-29°C annually. There are two main seasons; the rainy season which starts from June to around October and the dry season which starts from November to May. Malaria is endemic in this region and transmission occurs all year round with an average of 1-100 malaria cases per thousand per year[26].

The subjects for this study were taken from a study on thrombocytopenia and malaria (unpublished data). The study population consisted of patients consulting in 4 health units in Buea. These public health units include a hospital which is the main hospital in the area and 3 health centres. These units were chosen because they serve the majority of the population in the area due to their public nature and their location. Consecutive patients who came to each health unit for consultation were invited to participate in the study. Patients who satisfied all of the following characteristics were included in the study: fever (axillary temperature ≥37.5°C) or history of fever, age 18-65 years, and informed consent provided. Informed consent was obtained from all the participants in the study. Information about the study including possible risks and benefits was provided to the participants. Those who agreed to participate were asked to sign the consent form or provide a thumbprint. The study was approved by The Cameroon National Ethics Committee. A cross - sectional study was conducted from November 2007 to July 2008.

### Sample size

The subjects were taken from a study to identify thrombocytopenia as a risk factor for severe malaria in adults, for which the sample size was calculated. The prevalence of severe malaria in adults in the Province in 2006 is 35% (unpublished data). A 15% difference in both groups was considered of public health importance. The estimated sample size with 80% power and 5% significance level was 366.

### Data collection

Information on socio-demographic variables was collected through interview and was entered into a structured questionnaire. Other variables like rural residence, employment status, monthly income, marital status, educational status, use of insecticide treated nets (ITN) and use of antimalarials were collected through interview. The monthly income was used as a proxy for socioeconomic status. For all participants, blood samples were collected and sent to the laboratory.

The laboratory personnel were not aware of the clinical status of the patients. Thick blood films were prepared for all participants, air-dried, stained with Giemsa (Sigma, St. Louis, USA), and examined for malaria parasites by light microscopy. Parasites were detected using a binocular Olympus microscope (Olympus Optical Co., Ltd, Japan) under oil immersion. Parasites were counted against a minimum of 200 white blood cells for the determination of parasite density. Fifty high power fields (HPF) were scanned to confirm malaria negative slides. Thick blood films were examined for malaria parasites by two experienced microscopists. In case there was discordance in the results, that of the Kenya Medical Research Institute (KEMRI)-certified microcospist was re-examined by a senior scientist for approval. Quality control was ensured by a senior scientist who ensured that the authorised Standard operating procedure for thick blood film microscopy was strictly followed. Furthermore, the senior scientist randomly selected equal numbers of positive and negative slides for confirmation of results of malaria parasitaemia obtained.

Packed cell volume values were determined using a microhaematocrit centrifuge (HHC-24, Hanshin Medical CO., Ltd, Korea) according to the method described by Cheesbrough[27]. Briefly, plain microhaematocrit capillary tubes were filled with well mixed EDTA anticoagulated blood, leaving at least 15 mm of unfilled space. One end of the tube was sealed with cristaseal and the tubes centrifuged at 1200 rpm for 5 minutes in a microhaematocrit centrifuge. Packed cell volume was read off using a Hawksley microhaematocrit reader. Haemoglobin concentration (g/dl) was calculated from the PCV values, as described by Topley,[28] by dividing the PCV value by three. Anaemia was defined as haemoglobin levels less than 11 g/dl.

White Blood Cell (WBC), Red Blood Cell (RBC) and platelet counts were determined using a haemocytometer (improved Neubauer counting chamber) as described by Cheesbrough[27].

The Mean Corpuscular Volume (MCV) was calculated from the PCV and the RBC count values using the formula below:

### Data analysis

Data from the questionnaires was entered using the package EpiData version 3.0 (The EpiData association, Odense, Denmark). The data entered was checked for consistency and legal values. Data analysis was done using the package STATA version 8.2 (StataCorp, 4905 Lakeway Drive, College Station, Texas 77845 USA).

Baseline characteristics were compared in aparasitaemic and parasitaemic patients using means or proportions. Means were compared using the t-test or analysis of variance (ANOVA). In case of skewed data, the Mann-Whitney test was used. Proportions were compared using the chi-squared test. Quantitative variables were converted to categorical variables by splitting them into standard categories or tertiles. Univariate analysis was done by examining the effect of each measured variable on anaemia using the chi-squared test or the chi-squared test for trend. The T-test was used to compare the geometric means of parasite densities in both the anaemic and non-anaemic patients. The attributable risk fraction (ARF) was calculated by using the formula below[29]:

Multivariate analysis was done by entering each measured variable into a Logistic regression model. The variables were entered in a stepwise manner in order of the size of the effect (obtained from univariate analysis). The models were compared using the likelihood ratio test. A variable was dropped if there was no evidence (from p-value) that it fitted into the model or if the size of the effect of malaria parasitaemia on anaemia did not change substantially. A test for interaction among the categorical variables was also performed by comparing the model with and without the interaction term. The interaction term was also dropped if there was no evidence that it fitted into the model.

## Results

### Description of the study population

The study population consisted of a total of 250 adult patients, 68% (170/250) of the patients had a history of fever while 41.60% (104/250) had measured fever (axillary temperature ≥37.5°C) and 39.2% (98/250) had both history of fever and measured fever. The mean age (± SD) was 29.31 years ± 10.63 and 59.44% of the patients were females. The mean haemoglobin level in the study population was 13.16 g/dl ± 2.21 and the prevalence of anaemia (haemoglobin < 11 g/dl) was 14.80%. The median parasite count was 643 (range = 40 - 275 566) parasites per microlitre of blood and 25.60% of the patients had malaria parasitaemia. The reported use of insecticide treated nets was 16.95%. Patients with malaria parasitaemia were similar to those without malaria parasitaemia in terms of most of the baseline characteristics except for history of fever, temperature at enrolment and platelet count (Table 1).

**Table 1:** Baseline characteristics of study subjects

variable	Aparasitaemic		Parasitaemic		p-value*
		
	n	mean(SD)/%	n	mean(SD)/%	
age(years)	109	29.40(10.33)	52	29.12(11.35)	0.88

rural residence (%)	53	28.65	25	39.06	0.29

Sex (% female)	107	57.84	41	64.06	0.38

unemployed (%)	147	80.33	47	73.44	0.25

monthly income (thousand FCFA)	160	17.84(34.54)	54	18.06(34.45)	0.69^†^

married (%)	74	40.44	30	46.88	0.37

attained primary education (%)	54	30.17	14	21.88	0.46

use of ITN^b^(%)	30	17.14	10	16.39	0.89

use of antimalarial at home^a ^(%)	94	53.11	24	40.00	0.08

history of fever (%)	116	65.17	54	84.38	0.004

Temperature (°C)	170	37.35(0.75)	59	37.63(1.09)	0.03

white blood cell count (thousand per μl)	177	5.06(4.86)	64	5.03(2.39)	0.96

red blood cell count (million per μl)	177	4.28(0.85)	64	4.19(1.17)	0.51

mean corpuscular volume ( fl)	178	95.02(18.45)	64	96.45(26.88)	0.64

platelet count (thousand per μl)	177	154.25(53.12)	64	133.5(50.80)	0.007

means (SD) are presented for quantitative variables

*p-values compare estimates between aparasitaemic and parasitaemic subjects. In case of means, p-values are derived from the t-test. In case of percentages, p-values are obtained from the chi-squared test.

^†^obtained from the Mann-Whitney test

^a^use of antimalarial at home during the present fever episode

^b^ITN: Insecticide Treated Nets

### The effect of measured variables on anaemia

On univariate analysis, those with malaria parasitaemia had more anaemia than the aparasitaemic patients although this effect was not statistically significant (OR = 1.98, 95% CI = 0.94-4.17, p = 0.06) - Table 2. The proportion of subjects with anaemia in the parasitaemic patients was 14/64(21.88%) compared to 23/186(12.37%) in the aparasitaemic patients giving an attributable risk fraction of 43.46% [(21.88-12.37)/21.88] in those with malaria parasitaemia and 16.87% [(14.80-12.37)/12.37] in the study population. The geometric mean (± SD) malaria parasite density in those with anaemia was 1406.51 ± 10.63 parasites per microlitre while those with normal haemoglobin had a geometric mean (± SD) parasite density of 1003.44 ± 11.89 parasites per microlitre. Similarly, this difference in parasite densities was not statistically significant (p = 0.65). Those who lived in rural areas were more likely to have anaemia compared to those who lived in urban areas (p < 0.001). There was enough evidence that women had higher rates of anaemia compared to the men (p = 0.001). Those with missing values for age had lower proportion of malaria parasitaemia compared to the other subjects-13.48% versus 32.30% respectively (Chi-square = 10.65, p = 0.01).

**Table 2:** The effect of each measured variable on anaemia(Univariate analysis)

variable	N(n)*	OR(95% CI)^‡^	p-value^||^
malaria parasitaemia			
aparasitaemic	186(23)		
parasitaemic	64(14)	1.98(0.94-4.17)	0.06

age (years)			
<25	68(10)		
25-29	35(8)	1.72(0.60-4.89)	0.30
>30	58(9)	1.07(0.40-2.84)	0.90

residence			
urban	156(15)		
rural	78(22)	3.69(1.75-7.81)	<0.001

sex			
male	101(6)		
female	148(31)	4.20(1.64-10.71)	0.001

employment status			
employed	53(9)		
unemployed	194(28)	0.82(0.36-1.88)	0.65

monthly income (thousand FCFA)			
<15	148(24)		
≥15	66(7)	0.62(0.25-1.54)	0.30

marital status			
married	104(20)		
unmarried	143(17)	0.57(0.28-1.15)	0.11

educational status			
none	23(2)	0.44(0.09-2.19)	0.31
primary^+^	68(12)		
secondary	96(17)	1.00(0.44-2.27)	0.99
university	56(6)	0.56(0.19-1.62)	0.28

use of ITN			
yes	40(7)		
no	196(27)	0.75(0.30-1.88)	0.54

use of antimalarial at home			
yes	118(21)		
no	119(16)	0.72(0.35-1.46)	0.36

history of fever			
yes	170(30)		
no	72(7)	0.50(0.21-1.21)	0.12

temperature (T) (°C)			
<37.5	124(16)		
37.5 ≤ T < 38	56(12)	1.84(0.80-4.24)	0.15
≥38	48(7)	1.15(0.44-3.01)	0.77

white blood cell count (thousand/μl)			
<3.7	76(19)		
3.7-5	82(8)	0.32(0.13-0.81)	0.01
>5	83(10)	0.41(0.17-0.97)	0.04

platelet count (thousand per μl)			
<120	69(11)		
120 ≤ platelet < 150	65(10)	0.96(0.38-2.44)	0.93
≥150	107(15)	0.86(0.37-2.01)	0.73

*N = number of subjects in category, n = number with anaemia

^‡ ^crude odds ratios and confidence intervals

^|| ^p-value is obtained from the chi-square test or the chi-square test for trend

^+^Primary group was used as the baseline

### The effect of malaria parasitaemia on anaemia (logistic regression)

After adjusting for age, sex, rural residence, socioeconomic status(SES), antimalarial use and WBC count, the effect of parasitaemia on anaemia was more pronounced and this was of borderline significance (OR = 4.13, 95% CI = 1.20-14.23, p = 0.03).

After adding the variable ITN use in the model, the effect was even more pronounced and was statistically significant (OR = 4.33, 95%CI = 1.21-15.43, p = 0.02)-Table 3. This means there was evidence that those who had malaria parasites were about four times more likely to be anaemic compared to those without malaria parasites after adjusting for age, sex, rural residence, SES, use of antimalarials, ITN use and WBC count.

**Table 3:** The effect of each measured variable on anaemia (Logistic regression)

Variable	OR(95% CI)*	p-value^‡^
malaria parasitaemia		
aparasitaemic		
parasitaemic	4.33(1.21-15.43)	0.02

age(years)	1.03(0.97-1.09)	0.30

residence		
urban		
rural	5.13(1.57-16.78)	0.007

sex		
male		
female	8.83(1.83-42.62)	0.007

monthly income (thousand FCFA)		
<15		
≥15	1.64(0.46-5.86)	0.44

use of ITN		
yes		
no	2.19(0.43-11.08)	0.34

use of antimalarial at home		
yes		
no	0.39(0.11-1.38)	0.14

white blood cell count (thousand/μl)		
<3.7		
3.7-5	0.21(0.05-0.91)	0.04
>5	0.32(0.08-1.30)	0.11

*adjusted for each other variable in the model

^‡^p-values obtained from the Wald test the number of observations in the final model was 122

## Discussion

In this study, we sought to investigate the contribution of malaria in the prevalence of anaemia in adult febrile patients attending three principal health institutions in Buea, Cameroon.

Our findings indicate that anaemia and malaria parasitaemia are not rare in adult patients consulting in clinics in this setting where malaria is endemic and transmission is perennial. In high transmission areas like this, people usually experience frequent and repeated bites by mosquitoes and they develop immunity by the time they are adults. Adults usually have some level of parasites in them that do not necessarily cause clinical malaria but can lead to anaemia and its related complications. The proportion of individuals with malaria infection found in this population could partly be explained by the low reported use of insecticide treated nets. Our proportions are higher than that of a previous study conducted in a population of Mozambican adults[21].

The relationship between malaria parasitaemia and anaemia is well established in previous studies [4,11,13,15,17,18,20-23]. High levels of parasitaemia cause more destruction of red blood cells hence reducing haemoglobin levels leading to anaemia. Our results also indicate that malaria parasitaemia is responsible for about 44% of anaemia in those with malaria parasitaemia. This means if these patients are cleared of their parasites, this will reduce anaemia by more than one third. This information is useful for public health because the control of malaria can be targeted in order to reduce anaemia. This should however, be interpreted with caution because it assumes that there are no other factors which are responsible for anaemia (confounders). Policy on malaria control in non-pregnant adults should be geared towards reducing or clearing malaria parasites. However, there is conflicting opinion on the value of this kind of policy as it has been postulated that asymptomatic parasitaemia is useful in maintaining acquired immunity in adults while another school of thought disagrees with this hypothesis.

The main limitation of this study is confounding. Some potential confounders like helminth infections, micronutrient deficiencies and HIV infection were not measured. The study was conducted over a number of months which means the anaemia rates and even the parasite densities could change over time. However, the malaria transmission in this area is perennial and not seasonal. Human Chorionic Gonadotrophin (HCG) levels indicating the pregnancy status of the female patients was not measured which could affect the levels of haemoglobin. There were about 30% missing values for age which could cause insufficient adjustment for the confounding effect of age. This could have overestimated the effect of parasitaemia on anaemia since those with lower proportion of parasitaemias had missing values for age. The study may have been underpowered which could explain the borderline significance obtained. Microscopy was the only method used for the detection of malaria parasites meaning that some malaria negative patients could have had malaria parasites which could be detected by PCR and this could lead to underestimation of the effect of malaria parasitaemia on anaemia. However, microscopy is the method used in these health facilities for the management of patients and in this study, it was properly conducted using an authorised standard operating procedure, by well trained laboratory scientists.

## Conclusions

In adult patients in this setting with fever or history of fever, those with malaria parasitaemia are more likely to have anaemia and malaria parasitaemia has a great public health impact on anaemia. Public health interventions on malaria in non-pregnant adults should be geared towards clearing malaria parasites. Our results establish a baseline prevalence of malaria parasitaemia in adult patients (non-pregnant) with fever in this setting which will help clinicians in their differential diagnosis of malaria.

## Competing interests

The authors declare that they have no competing interests.

## Authors' contributions

ENT was involved in the conception and design of the study, analysis and interpretation of the data and drafting of the manuscript. EAA was involved in the analysis and interpretation of the data and drafting of the manuscript. PMN was involved in the analysis and interpretation of the data and drafting of the manuscript. All authors approved the final version of the manuscript.

## References

[B1] Calis JC Phiri KS Faragher EB Brabin BJ Bates I Cuevas LE de Haan RJ Phiri AI Malange P Khoka M Severe anemia in Malawian children N Engl J Med 2008 358 888 899 10.1056/NEJMoa072727 18305266

[B2] Koukounari A Fenwick A Whawell S Kabatereine NB Kazibwe F Tukahebwa EM Stothard JR Donnelly CA Webster JP Morbidity indicators of Schistosoma mansoni: relationship between infection and anemia in Ugandan schoolchildren before and after praziquantel and albendazole chemotherapy Am J Trop Med Hyg 2006 75 278 286 16896133

[B3] Hinderaker SG Olsen BE Lie RT Bergsjo PB Gasheka P Bondevik GT Ulvik R Kvale G Anemia in pregnancy in rural Tanzania: associations with micronutrients status and infections Eur J Clin Nutr 2002 56 192 199 10.1038/sj.ejcn.1601300 11960293

[B4] Muhangi L Woodburn P Omara M Omoding N Kizito D Mpairwe H Nabulime J Ameke C Morison LA Elliott AM Associations between mild-to-moderate anaemia in pregnancy and helminth malaria and HIV infection in Entebbe Uganda Trans R Soc Trop Med Hyg 2007 101 899 907 10.1016/j.trstmh.2007.03.017 17555783 PMC1950430

[B5] Van Eijk AM Ayisi JG Ter Kuile FO Misore AO Otieno JA Kolczak MS Kager PA Steketee RW Nahlen BL Malaria and human immunodeficiency virus infection as risk factors for anemia in infants in Kisumu western Kenya Am J Trop Med Hyg 2002 67 44 53 12363063 10.4269/ajtmh.2002.67.44

[B6] Mugisha JO Shafer LA Paal L Van der Mayanja BN Eotu H Hughes P Whitworth JA Grosskurth H Anaemia in a rural Ugandan HIV cohort: prevalence at enrolment incidence, diagnosis and associated factors Trop Med Int Health 2008 13 788 794 18384480 10.1111/j.1365-3156.2008.02069.x

[B7] Nielsen NO Simonsen PE Magnussen P Magesa S Friis H Cross-sectional relationship between HIV lymphatic filariasis and other parasitic infections in adults in coastal northeastern Tanzania Trans R Soc Trop Med Hyg 2006 100 543 550 10.1016/j.trstmh.2005.08.016 16324731

[B8] Stoltzfus RJ Chwaya HM Montresor A Albonico M Savioli L Tielsch JM Malaria, hookworms and recent fever are related to anemia and iron status indicators in 0- to 5-y old Zanzibari children and these relationships change with age J Nutr 2000 130 1724 1733 10867043 10.1093/jn/130.7.1724

[B9] Brooker S Peshu N Warn PA Mosobo M Guyatt HL Marsh K Snow RW The epidemiology of hookworm infection and its contribution to anaemia among pre-school children on the Kenyan coast Trans R Soc Trop Med Hyg 1999 93 240 246 10.1016/S0035-9203(99)90007-X 10492749

[B10] Sturrock RF Kariuki HC Thiongo FW Gachare JW Omondi BG Ouma JH Mbugua G Butterworth AE Schistosomiasis mansoni in Kenya: relationship between infection and anaemia in schoolchildren at the community level Trans R Soc Trop Med Hyg 1996 90 48 54 10.1016/S0035-9203(96)90477-0 8730312

[B11] Kagu MB Kawuwa MB Gadzama GB Anaemia in pregnancy: a cross-sectional study of pregnant women in a Sahelian tertiary hospital in Northeastern Nigeria J Obstet Gynaecol 2007 27 676 679 10.1080/01443610701612144 17999291

[B12] Menendez C Fleming AF Alonso PL Malaria-related anaemia Parasitol Today 2000 16 469 476 10.1016/S0169-4758(00)01774-9 11063857

[B13] Tarimo SD Appraisal on the prevalence of malaria and anaemia in pregnancy and factors influencing uptake of intermittent preventive therapy with sulfadoxine-pyrimethamine in Kibaha district Tanzania East Afr J Public Health 2007 4 80 83 18085136

[B14] Achidi EA Kuoh AJ Minang JT Ngum B Achimbom BM Motaze SC Ahmadou MJ Troye-Blomberg M Malaria infection in pregnancy and its effects on haemoglobin levels in women from a malaria endemic area of Fako Division, South West Province Cameroon J Obstet Gynaecol 2005 25 235 240 10.1080/01443610500060628 16147724

[B15] Adam I Khamis AH Elbashir MI Prevalence and risk factors for anaemia in pregnant women of eastern Sudan Trans R Soc Trop Med Hyg 2005 99 739 743 10.1016/j.trstmh.2005.02.008 16024057

[B16] Mockenhaupt FP Mandelkow J Till H Ehrhardt S Eggelte TA Bienzle U Reduced prevalence of Plasmodium falciparum infection and of concomitant anaemia in pregnant women with heterozygous G6PD deficiency Trop Med Int Health 2003 8 118 124 10.1046/j.1365-3156.2003.01008.x 12581435

[B17] Rogerson SJ Broek NR van den Chaluluka E Qongwane C Mhango CG Molyneux ME Malaria and anemia in antenatal women in Blantyre, Malawi: a twelve-month survey Am J Trop Med Hyg 2000 62 335 340 11037774 10.4269/ajtmh.2000.62.335

[B18] Huddle JM Gibson RS Cullinan TR The impact of malarial infection and diet on the anaemia status of rural pregnant Malawian women Eur J Clin Nutr 1999 53 792 801 10.1038/sj.ejcn.1600851 10556986

[B19] Shulman CE Graham WJ Jilo H Lowe BS New L Obiero J Snow RW Marsh K Malaria is an important cause of anaemia in primigravidae: evidence from a district hospital in coastal Kenya Trans R Soc Trop Med Hyg 1996 90 535 539 10.1016/S0035-9203(96)90312-0 8944266

[B20] Ouma P van Eijk AM Hamel MJ Parise M Ayisi JG Otieno K Kager PA Slutsker L Malaria and anaemia among pregnant women at first antenatal clinic visit in Kisumu western Kenya Trop Med Int Health 2007 12 1515 1523 18076560 10.1111/j.1365-3156.2007.01960.x

[B21] Mayor A Aponte JJ Fogg C Saute F Greenwood B Dgedge M Menendez C Alonso PL The epidemiology of malaria in adults in a rural area of southern Mozambique Malar J 2007 6 3 10.1186/1475-2875-6-3 17233881 PMC1796885

[B22] Asobayire FS Adou P Davidsson L Cook JD Hurrell RF Prevalence of iron deficiency with and without concurrent anemia in population groups with high prevalences of malaria and other infections: a study in Cote d'Ivoire Am J Clin Nutr 2001 74 776 782 11722959 10.1093/ajcn/74.6.776

[B23] Akhwale WS Lum JK Kaneko A Eto H Obonyo C Bjorkman A Kobayakawa T Anemia and malaria at different altitudes in the western highlands of Kenya Acta Trop 2004 91 167 175 10.1016/j.actatropica.2004.02.010 15234666

[B24] Koram KA Owusu-Agyei S Fryauff DJ Anto F Atuguba F Hodgson A Hoffman SL Nkrumah FK Seasonal profiles of malaria infection anaemia, and bednet use among age groups and communities in northern Ghana Trop Med Int Health 2003 8 793 802 10.1046/j.1365-3156.2003.01092.x 12950665

[B25] Carneiro IA Smith T Lusingu JP Malima R Utzinger J Drakeley CJ Modeling the relationship between the population prevalence of Plasmodium falciparum malaria and anemia Am J Trop Med Hyg 2006 75 82 89 16931819 10.4269/ajtmh.2006.75.82

[B26] WHO World Malaria Report 2008 WHO/HTM/GMP/20081 2008 Geneva, Switzerland 1 56

[B27] Cheesbrough M District Laboratory Practice in Tropical Countries. Part2 2000 Cambridge: University Press

[B28] Topley E Anaemia in rural Africa: community support for control activities where malaria is common 1998 Cambridge: FSG MediMedia

[B29] Hennekens C Buring J Epidemiology in Medicine 1987 Boston/Toronto: Little, Brown

